# 3D-Printed Personal Protective Face Shields During the COVID-19 Pandemic: A Survey of Canadian Frontline Workers

**DOI:** 10.7759/cureus.18141

**Published:** 2021-09-20

**Authors:** Anser Daud, Isra M Hussein, Peter Chengming Zhang, Yousuf Ahmed, Jessica Trac, Nina Vujovic, Syed F Rizvi, Paul R Kuzyk

**Affiliations:** 1 Faculty of Medicine, University of Toronto, Toronto, CAN; 2 Granovsky Gluskin Division of Orthopaedic Surgery, Sinai Health System, Toronto, CAN; 3 Leslie Dan Faculty of Pharmacy, University of Toronto, Toronto, CAN; 4 Rotman School of Management, University of Toronto, Toronto, CAN; 5 Michael G. DeGroote School of Medicine, McMaster University, Hamilton, CAN; 6 Division of Orthopaedic Surgery, University of Toronto, Toronto, CAN

**Keywords:** cross-sectional survey, real-world survey, ppe strategies, ppe shortage, ‎3d printing, survey research, infection prevention and control, frontline workers, personal protective equipment (ppe), three-dimensional (3d) printing

## Abstract

Background

During the coronavirus disease 2019 pandemic, three-dimensional (3D) printing was utilized to rapidly produce face shields for frontline workers in response to an acute shortage of personal protective equipment (PPE). In this study, we examine the perceived utility and performance of 3D-printed (3DP) face shields through a survey of frontline workers in Ontario, Canada.

Methodology

Frontline workers who received community-produced 3DP face shields from the Canadian initiative “3DPPE GTHA” (March-December 2020) were invited to participate in the study. The survey response rate was 54.3%. Of 63 respondents, 39 were patient-facing and 24 were community-facing frontline workers. Participants were asked to rate performance measures in 10 categories on a five-point Likert scale. Data were categorized by organization and frontline worker type, and a t-test was used to determine statistically significant differences among subgroups.

Results

The mean preference for 3DP face shields among respondents was 3.2 out of 5 (95% confidence interval [CI]: 2.1-4.3). Community-facing respondents reported significantly greater overall utility scores for 3DP face shields (3.58, 95% CI: 3.38-3.79) compared to respondents working in a patient-facing profession (2.95, 95% CI: 2.77-3.13; p < 0.05). However, no differences were reported in portability and compatibility with other PPE. Respondents from organizations with large service volumes reported significantly lower overall utility scores (2.67, 95% CI: 2.44-2.89) than respondents in organizations with smaller service volumes (3.45, 95% CI: 3.28-3.62; p < 0.05).

Conclusions

Community-facing frontline workers and those from smaller service volume organizations endorse higher utility for 3DP face shields than patient-facing frontline workers. Despite this, frontline workers generally rate 3DP face shields positively. 3DP face shields are a viable option for personal and community use and can be used to supplement supply in a community setting.

## Introduction

The emergence of the coronavirus disease 2019 (COVID-19) pandemic caused a sharp increase in the demand for medical resources on healthcare systems globally [1]. In Canada, rapidly increasing caseloads had impending downstream effects on healthcare systems and communities at large. This, coupled with disrupted supply chains, led to a sudden, acute shortage of supplies and, notably, personal protective equipment (PPE). Among the most urgent concerns was the need to provide adequate protection for high-risk groups such as frontline healthcare workers, for whom PPE is the most significant primary prevention from COVID-19 [2]. Scarcity of PPE during the early months of the COVID-19 pandemic led to rationing and recycling [3]. In the midst of this unexpected challenge, there arose an opportunity for grassroots initiatives to augment the production of PPE through the use of three-dimensional (3D) printing technology [4].

3D printing can enable rapid production of lightweight frameworks, such as face shields, ear savers, stethoscopes, and masks, with high levels of precision and design customization [5-7]. With access to open-source data, models that have been tested and approved can be utilized or modified to meet the needs of the users. The most common 3D-printed (3DP) face shield designs are universally sized and consist of two easy-to-assemble pieces [8,9]. Within Canada, the 3DPPE GTHA initiative was the largest not-for-profit program supplying over 25,000 3DP face shields to frontline organizations across the province of Ontario [10]. In this study, we examine the perceived utility of the 3DP face shields supplied by this initiative, with a focus on 10 categories, as reported through evaluations from frontline workers who received 3DP face shields. To the best of our knowledge, this study is the largest in the literature examining the perceived utility of 3DP face shields by a broad range of frontline workers and provides future direction for the development, production, and distribution of 3DP PPE.

## Materials and methods

The methodology was informed by the Checklist for Reporting Results of Internet E-Surveys (CHERRIES) guidelines and peer-reviewed guides on the assessment of the quality of online surveys (Appendix A) [11,12]. Ethics approval was obtained from the University of Toronto’s Research Ethics Board, Protocol #23085. Participants were informed of the study details, potential risks and benefits, confidentiality information, and their right to withdraw from participation. Written documentation of informed consent was completed by participants through an online form prior to survey completion. Survey responses were deidentified and separated from participant names.

Eligibility criteria and recruitment

Frontline workers who utilized 3DP face shields provided by 3DPPE GTHA for patient care or workplace safety from March to December 2020 were invited by email to participate in the research survey. From a decentralized collection of over 300 contacts, a total of 116 patient-facing and community-facing frontline workers were screened and selected by the study team based on the likelihood of response (i.e., known responders versus central inboxes) and heterogeneity of respondents. Attempts to reach participants were made up to three times.

Study design

**Figure 1:**
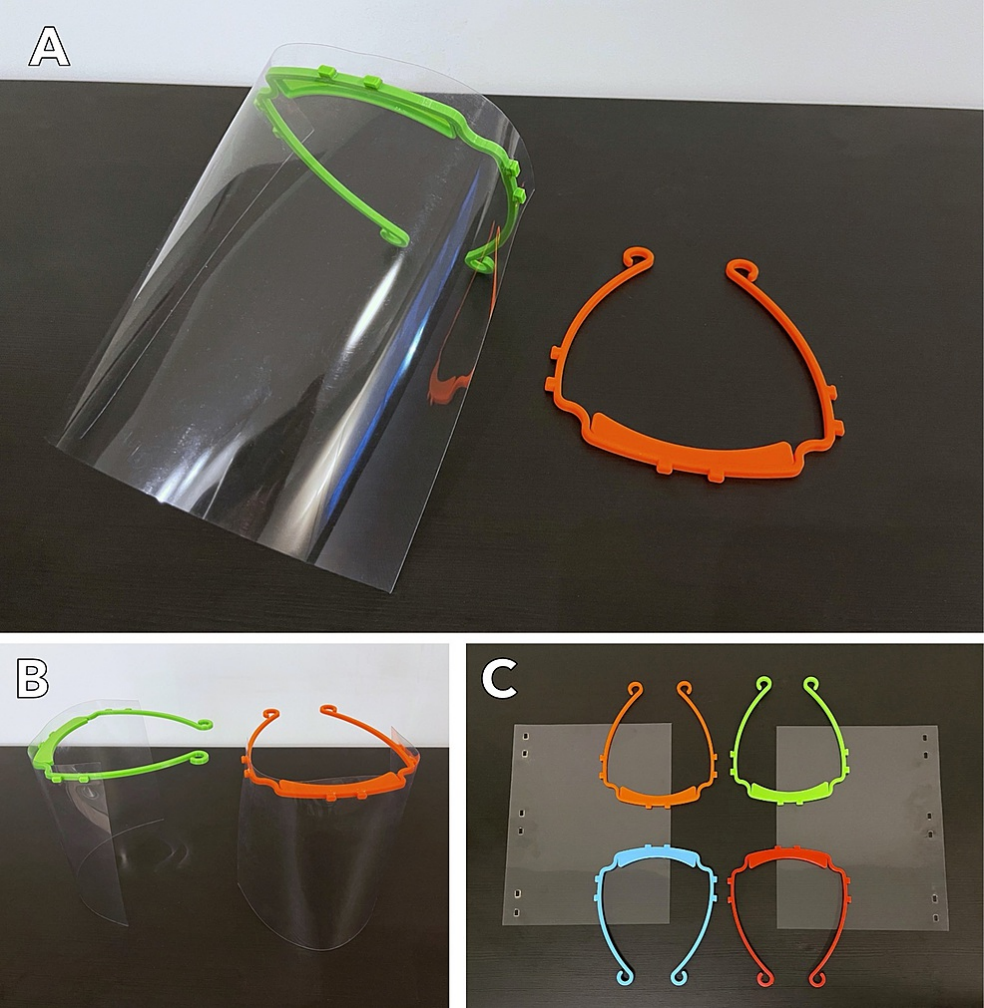
The 3DP face shield as produced by the 3DPPE GTHA initiative. The face shield, pictured in assembled and unassembled formats, was distributed to nearly 25,000 frontline workers across Canada. 3DP: 3D printed 3DPPE GTHA: A grassroots, not-for-profit initiative focused on producing and distributing 3D-printed face shields to Canadian healthcare workers.

**Figure 2:**
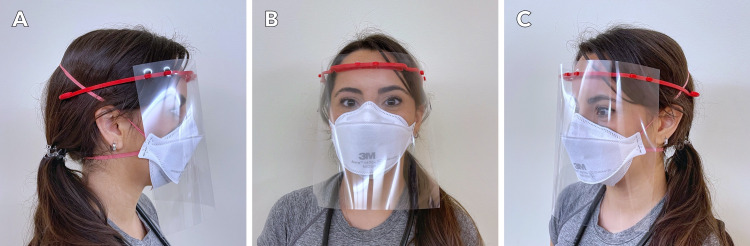
A Canadian frontline healthcare worker wearing a fully assembled 3DP face shield in combination with a face mask. 3DP: 3D printed Photo courtesy of Ida Alizadeh and Anser Daud under creative commons license CC BY-NC-ND 4.0.

**Figure 3:**
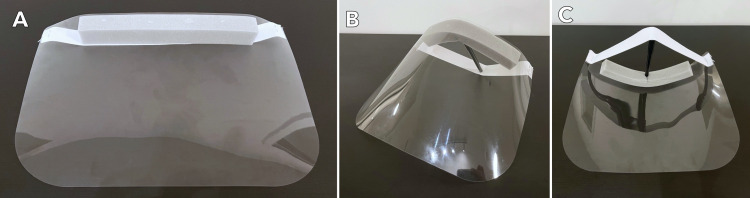
Multiple views of a “conventional” face shield, as defined by the study authors to survey recipients.

Study participants had the opportunity to fill an online survey exploring their evaluation of the efficacy of 3DP face shields (Figures 1, 2) compared to conventional face shields (Figure 3) from December 20, 2021 to March 31, 2021 (Appendix B). The survey collected information about the work setting of respondents, including the type of facility, the number of healthcare workers within the organization, healthcare discipline, and volume of service. Items pertaining to the utility of 3DP face shields were generated by the committee, by the authors. Similar categories were combined and categories deemed irrelevant were dropped in consultation with the senior author (PRK). Survey items included 10 distinct characteristics such as comfort, visibility, durability, and protectiveness, as well as the cumulative assessment of preference for 3DP face shields compared to a conventional face shield (Table 1, Appendix C). Each item was rated on a Likert scale from 1 (worst) to 5 (best) on the survey scale with an option to answer n/a on any item.

**Table 1:** Survey categories for the evaluation of 3DP face shields. 3DP: 3D printed; PPE: personal protective equipment

Survey categories
Comfort
Stability
Visibility
Durability
Protectiveness
Ease of assembly
Portability
Compatibility with other PPE
Compatibility with sterile field
Ability to communicate
Preference (1 = conventional preferred, 3 = equal preference, 5 = 3DP preferred)

Analysis

Means and 95% confidence intervals (95% CI) were calculated for each survey category outcome. The anonymized data collected from the survey were categorized by organization type, organization size, and worker type. Subgroups were defined as medical (i.e., patient-care oriented) versus nonmedical (i.e., community) organizations, large (i.e., organizations serving >200 clients/day) versus small organizations, and patient-facing versus community-facing frontline workers. A t-test was conducted to determine statistically significant differences in the subgroup analysis. A p-value of <0.05 was considered statistically significant. All data analysis was carried out using the StatTools software (Version 8.1, New York, United States). Incomplete responses were excluded from the dataset. There were no missing data.

## Results

A total of 116 selected frontline workers from the 3DPPE GTHA recipient database were recruited with personalized email invitations to complete the survey. The overall survey response rate was 56.0%. Two incomplete responses (i.e., less than 20% of survey questions answered) were removed from the final analysis, resulting in a total of 63 unique responses to the survey and a final response rate of 54.3%.

Demographic characteristics of respondents

All 63 respondents were grouped into seven major organization types and nine major frontline worker types (Table 2). Organizations consisted of hospitals (n = 10), food banks (n = 7), outpatient medical clinics (n = 9), educational or day-care facilities (n = 6), dental clinics (n = 10), and first responders (n = 1). Frontline workers were grouped into medical frontline workers (n = 30) and community frontline workers (n = 33). Patient-facing frontline workers consisted of medical professionals such as physicians, nurses, pharmacists, dentists, and therapists. Community-facing frontline workers included those working in a community service environment not constituting patient care such as food banks, daycares, or shelters. Over half of all organizations (n = 34, 54%) had a client volume of 10 to 50 patients per day, while fewer had volumes of greater than 50 clients per day (n = 21, 33.3%) or less than 10 clients per day (n = 8, 12.7%).

**Table 2:** Demographic characteristics of the respondents.

	n	%
Respondents	63	100.00%
Organization types		
Hospital	10	15.87%
Foodbank	7	11.11%
Outpatient medical clinic	9	14.29%
Educational or daycare facility	6	9.52%
Long-term cares	20	31.75%
Dental clinic	10	15.87%
First responders	1	1.59%
Frontline worker types		
Physicians	9	14.29%
Nurses	11	17.46%
Foodbank staff	6	9.52%
Educational	5	7.94%
Long-term care staff	14	22.22%
Dentists	10	15.87%
Pharmacists	3	4.76%
Shelter staff	4	6.35%
Paramedic	1	1.59%
Patient/Client service volumes		
1–10 per day	8	12.70%
10–50 per day	34	53.97%
50–200 per day	18	28.57%
200+ per day	3	4.76%

Mean survey results

**Figure 4:**
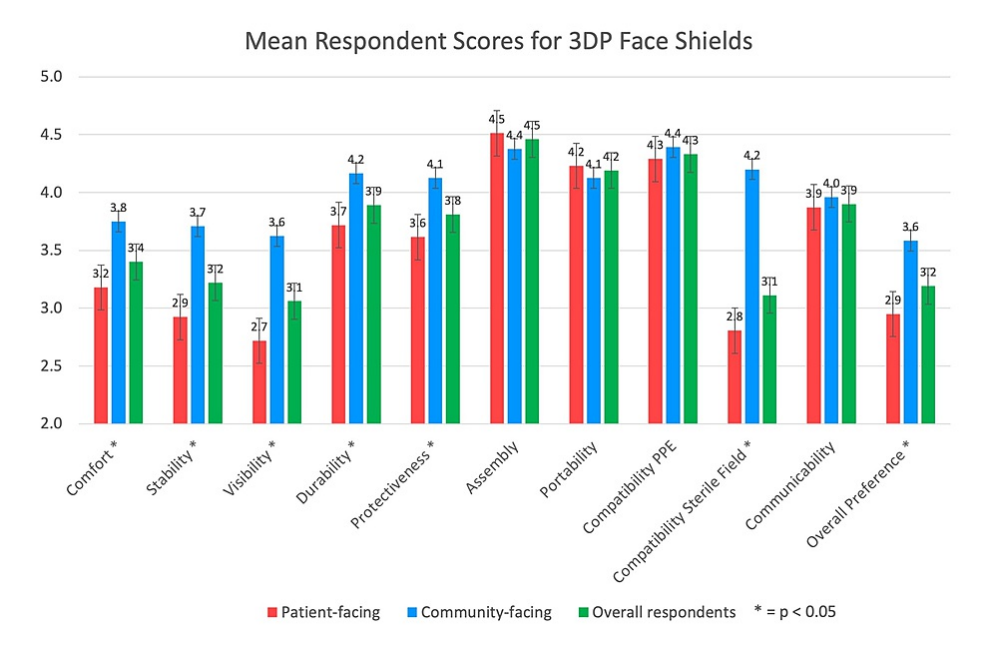
Mean respondent scores are demonstrated by category. Graphs contrasting 3DP face shield scores by overall, patient-facing, and community-facing frontline workers are visualized side by side. Red: patient-facing frontline workers; blue: community-facing frontline workers; green: overall respondents. * p-value < 0.05 was considered significant. 3DP: 3D printed

The mean scores of survey respondents for comfort, stability, visibility, durability, protectiveness, ease of assembly, portability, compatibility, ability to communicate, and overall preference are demonstrated in Figure 4 and Table 3. The mean preference for 3DP face shields among all respondents was 3.2 (95% CI: 2.1-4.3). The highest mean score for all respondents was 4.5 (95% CI: 3.8-5) for ease of assembly. The lowest mean score for all respondents was 3.1 (95% CI: 1.8-4.4) for visibility.

**Table 3:** Mean ratings for survey variables. CI: confidence interval; PPE: personal protective equipment; 3DP: 3D printed

	Overall mean	95% CI	n
Comfort	3.40	2.5–4.3	63
Stability	3.22	2.1–4.4	63
Visibility	3.06	1.8–4.4	63
Durability	3.89	3.1–4.7	63
Protectiveness	3.81	2.8–4.8	63
Ease of assembly	4.46	3.8–5	63
Portability	4.19	3.4–5	63
Compatibility other PPE	4.33	3.7–5	61
Compatibility sterile field	3.11	1.7–4.5	46
Ability to communicate	3.90	3.0–4.8	63
Preference (1 = conventional preferred, 3 = equally preferred, 5 = 3DP preferred)	3.19	2.1–4.3	63

Subgroup analyses

Frontline workers from nonmedical organizations rated 3DP face shields more positively than frontline workers from medical organizations. Respondents from medical organizations found 3DP face shields inferior to conventional face shields in several categories, including comfort, stability, visibility, and sterile field compatibility (p < 0.05) and reported an overall preference toward conventional face shields compared to workers from nonmedical organizations (Table 4). The overall preference of 2.7 (95% CI: 2.54-2.86) for respondents from medical organizations was significantly lower than that of respondents from nonmedical organizations at 3.64 (95% CI: 3.44-3.83; p < 0.05).

**Table 4:** Subgroup analysis of ratings by frontline workers categorized by the organization type of frontline workers (i.e., healthcare or nonhealthcare). *p-value < 0.05 was considered significant. PPE: personal protective equipment; 3DP: 3D printed

	Medical mean (n = 30)	Nonmedical Mean (n = 33)	P-value
Comfort	2.93	3.82	<0.0001*
Stability	2.57	3.82	<0.0001*
Visibility	2.27	3.79	<0.0001*
Durability	3.73	4.03	0.1536
Protectiveness	3.43	4.15	0.0063*
Assembly	4.57	4.36	0.2439
Portability	4.23	4.15	0.668
Compatibility other PPE	4.27	4.39	0.4545
Compatibility sterile field	2.45	4.24	<0.0001*
Ability to communicate	3.73	4.06	0.1318
Preference (1 = conventional preferred, 3 = equally preferred, 5 = 3DP preferred)	2.70	3.64	0.0005*

Frontline Workers From Smaller Service Volume Organizations Rated 3DP Face Shields More Positively Than Larger Service Volume Counterparts

Frontline workers from large-volume organizations, which included hospitals and long-term care homes, had an overall preference of 2.67 (95% CI: 2.44-2.89) for 3DP face shields, which was significantly lower than 3.45 (95% CI: 3.28-3.62) as reported by respondents from smaller-volume organizations, which included outpatient clinics, pharmacies, and daycare facilities. Frontline workers from smaller organizations reported 3DP shields to be more favorable in terms of communication (p < 0.05) and protectiveness (p < 0.05), while frontline workers from larger organizations found shields easier to assemble compared to those from smaller organizations (p < 0.05) (Table 5).

**Table 5:** Subgroup analysis of ratings by frontline workers categorized by small- and large-volume service organizations. Large organizations were defined as those serving >200 clients/day, while small organizations were defined as those serving 200 or fewer clients per day. *p-value < 0.05 was considered significant. PPE: personal protective equipment; 3DP: 3D printed

	Large organizations (n = 21)	Small organizations (n = 42)	P-value
Comfort	3.2857	3.4524	0.4714
Stability	2.905	3.381	0.1333
Visibility	3.143	3.024	0.734
Durability	3.5714	4.0476	0.0244
Protectiveness	3.095	4.167	0.0007*
Assembly	4.8095	4.2857	0.0006*
Portability	4.1905	4.1905	1
Compatibility other PPE	4.25	4.3659	0.4746
Compatibility sterile field	2.889	3.25	0.3995
Ability to communicate	3.4762	4.119	0.0022*
Preference (1 = conventional preferred, 3 = equally preferred, 5 = 3DP preferred)	2.667	3.452	0.0072*

Community-Facing Frontline Workers Found 3DP Face Shields Superior Compared to Patient-Facing Frontline Workers

Patient-facing respondents, who were categorized by their profession, included physicians, pharmacists, nurses, paramedics, and dentists. Staff or volunteers in daycare facilities, shelters, or food banks were considered community-facing as they primarily interacted with healthy individuals. The overall preference for respondents employed in patient-facing professions was 2.95 (95% CI: 2.77-3.13), which was significantly lower than respondents from nonpatient-facing professions at 3.58 (95% CI: 3.38-3.79). T-test analysis revealed that patient-facing respondents found 3DP face shields to be less preferred than conventional face shields compared to community-facing respondents (p < 0.05) (Table 6). Community-facing respondents had significantly higher ratings for comfort, stability, visibility, durability, and protectiveness (p < 0.05).

**Table 6:** Subgroup analysis of ratings by frontline worker type categorized as patient-care-oriented or community-oriented. * p-value < 0.05 was considered significant. PPE: personal protective equipment; 3DP: 3D printed

	Patient facing (n = 39)	Community facing (n = 24)	P-value
Comfort	3.1795	3.75	0.015*
Stability	2.923	3.708	0.0047*
Visibility	2.718	3.625	0.0055*
Durability	3.7179	4.1667	0.0209*
Protectiveness	3.615	4.125	0.0235*
Assembly	4.5128	4.375	0.4355
Portability	4.2308	4.125	0.6216
Compatibility other PPE	4.2895	4.3913	0.5308
Compatibility sterile field	2.806	4.2	<0.0001*
Ability to communicate	3.8718	3.9583	0.6932
Preference (1 = conventional preferred, 3 = equally preferred, 5 = 3DP preferred)	2.949	3.583	0.0249*

## Discussion

3D printing technology has been increasingly used during the COVID-19 pandemic to produce medical supplies including PPE. Early in the pandemic, there was an increased focus on innovative but simple designs for 3DP face shields among researchers across the globe. Several of these designs were shared on open-source communities and eventually underwent review by the National Institutes of Health for use in the clinical setting [8,9]. The 3DPPE GTHA initiative opted to produce the popular 3DVerkstan face shield design and distributed face shields to healthcare facilities across Ontario, Canada [9,10]. While the use of 3DP face shields by frontline workers has increased exponentially, there remains a paucity of literature evaluating utility outcomes of 3DP PPE to date. One study by Wierzbicki et al. has examined the use of 3D printing to rapidly produce various open-source face shields for healthcare workers [5,6]. Sapoval et al. adapted the open-source PRUSA design and investigated the use of the face shield in a procedural setting, assessing for visual comfort and tolerance among interventional radiologists [13]. The authors found good acceptance of 3DP face shields within their realm of practice. The present study is the largest in the literature reporting on the broad utility of 3DP face shields about 10 outcomes, as rated by a range of Canadian frontline workers.

Professions that routinely require personal protective equipment preferred conventional face shields

Our results demonstrate that 3DP face shields were perceived more positively by community-facing frontline workers than patient-facing frontline workers (Tables 4, 6). For example, there were significantly higher ratings by community-facing workers in categories of comfort, stability, protectiveness, and visibility. These findings may be attributable to the familiarity medical workers have with conventional face shields due to their routine use of this type of equipment before the COVID-19 pandemic. As such, the expectations of clinical workers may have been higher, or they may have been more alert to how 3DP models deviate from conventional face shields. Additionally, there may be differing expectations for the level of protectiveness required from the PPE medical frontline workers utilized as a result of the level of COVID-19 transmission risk associated with their roles. Differing expectations of the level of protection provided by PPE are exacerbated by the stress healthcare workers experience amid uncertainty about the mode of transmission of severe acute respiratory syndrome coronavirus 2 and fear due to lack of industry-grade PPE [14,15]. These perceptions may explain the higher preference for 3DP face shields by community-facing frontline staff and nonmedical organizations compared to their clinical counterparts. Smaller service volume organizations provided lower ratings for ease of assembly, which is an unexplained finding, but it remains possible that larger organizations such as hospitals had personnel available to help distribute and assemble face shields.

Considerations and barriers to implementation

3DP PPE may help meet individual or local needs during supply shortages. During the COVID-19 pandemic, various grassroots initiatives successfully mobilized individuals of varying skill levels, from academics and professionals to hobbyists, to provide thousands of 3DP face shields to healthcare facilities and organizations. The print job can be completed with standard, cost-friendly printers and available open-source designs such as the one assessed in this study (i.e., 3DVerkstan) [9]. Printing open-source designs requires some computer literacy but not extensive technical or design expertise. 3DP face shields are also comparatively more simple to print than complex designs such as nasal swabs [16]. With access to 3D printers, individuals and organizations would have the capacity to rapidly prototype and manufacture tens to hundreds of face shields on demand and reduce lag time for organizations to access PPE during times of high strain on supply chains.

While 3D printers are cheap and readily available, the inability to complete quality testing and safety assessment remains a barrier to mainstream use. In comparison to conventional face shields, 3DP face shields do not undergo rigorous testing to ensure adequate droplet protection. Current 3D printing technology is most suitable for rapid prototyping and assessing the feasibility of proof-of-concept designs as opposed to manufacturing large volumes of prints. Therefore, 3D printing alone would not suffice in meeting the demands of PPE for large organizations, which may require hundreds of new face shields daily. Given these challenges, and the differences in rating between larger, clinical versus smaller community organizations, we find that smaller and independent community-facing organizations with a fixed need for PPE will benefit most from the use of 3DP face shields.

Limitations

This study had several limitations. First, the results of this survey relate to one particular 3DP face shield model, 3DVerkstan, distributed to frontline workers in Ontario, Canada. Although the most common designs are similar in concept, some outcomes may not be generalizable to other 3DP models. Second, our survey population is not a random sample as those surveyed were particularly recipients of the 3DPPE GTHA face shield donations who received donations upon request. Third, without data on specific professions and work duties (i.e., professional subspecialties and exposure), a multitude of frontline workers were self-identified and ultimately categorized upon the conclusion of the data collection phase by researchers into broad groups such as “medical” versus “nonmedical.” This classification was necessary as the study analysis required a broad distinction between the two groups. Despite these limitations, this study is one of very few in the current literature [2,3] reporting on outcomes of frontline workers using 3DP face shields.

## Conclusions

The community response toward COVID-19 has yielded an increase in the use of 3D printing to produce PPE. While numerous studies have reported the use of 3D printing for PPE, the present study is the largest in the literature survey of frontline workers demonstrating that, in general, nonmedical frontline workers and organizations appraised 3DP face shields more favorably than medical frontline workers. Reasons for this may include differing expectations for levels of protectiveness and comfort, as well as prior familiarity with conventional equipment. Relatively higher utility ratings from community-facing frontline workers, and potential barriers to high-volume, quality-controlled 3D printing, suggest that 3DP face shields are most suitable for use in independent, community organization settings. Furthermore, during potential shortages of PPE, an effective strategy may involve the distribution of community-manufactured 3DP face shields to nonclinical organizations while conventional face shields are allocated to hospitals and other large clinical facilities. Further study in this area may provide insights on optimizing the utility of community contributions.

## Figures and Tables

**Figure 5:**
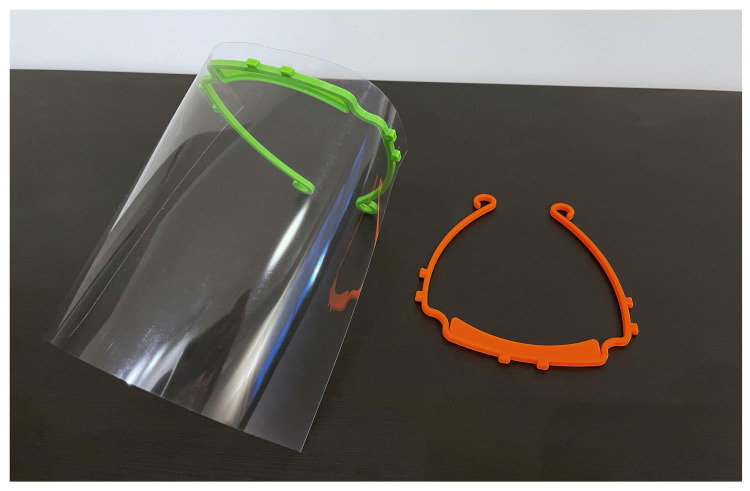
3DP face shield. 3DP: 3D printed

**Figure 6:**
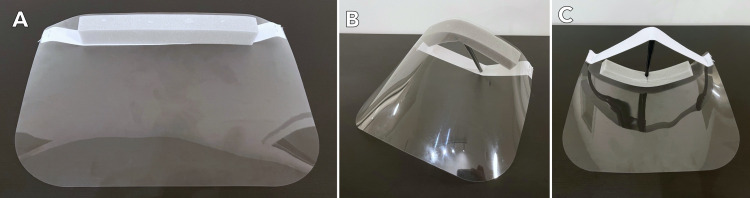
Conventional face shield.

## Appendices

Appendix A: Checklist for Reporting Results of Internet E-Surveys (CHERRIES)

**Table 7:** Checklist for Reporting Results of Internet E-Surveys. 3DPPE GTHA: A grassroots, not-for-profit initiative focused on producing and distributing 3D-printed face shields to Canadian healthcare workers. This checklist has been modified from Eysenbach G. Improving the quality of web surveys: the Checklist for Reporting Results of Internet E-Surveys (CHERRIES). J Med Internet Res. 2004, 6:e34 [erratum in J Med Internet Res. 2012, 14:e8] [12].

Checklist item	Explanation	Page number
Describe survey design	Frontline workers who were provided 3D-printed face shields between March and December 2020 were invited to complete the survey	3
IRB approval	Ethics approval was obtained from the University of Toronto’s Research Ethics Board	3
Informed consent	As part of the informed consent process, participants told the length of time of the survey, which data were stored (i.e., survey responses were detached and stored separately from informed consent data), where and for how long the data would be maintained, who the investigators are, and the purpose of the study	3
Data protection	No personal information was collected and respondents did not have to provide identifying features other than their profession	3
Development and testing	The survey was developed using Google Forms and the electronic questionnaire had been internally tested by researchers before fielding the questionnaire	N/A
Open survey versus closed survey	A closed survey was used where only select respondents were able to access the survey. However, the link could be shared as it was not password protected as there may be multiple participants snowballed at one site	3
Contact mode	The initial contact was made through email where respondents were informed of the survey and provided the link to access the survey	3
Advertising the survey	The survey was disseminated to recipients of the 3DPPE GTHA initiative via an email invitation to participate and a link to the survey. A list of 3D-printed face shield donation contacts was obtained from the initiative’s database. The recipients were primary contacts who the 3DPPE GTHA initiative was in touch with to facilitate the donation of face shields. If contacts themselves were frontline workers, they were asked to complete the survey themselves in addition to passing the survey on to colleagues. If the contacts were administered staff, they were asked to pass the survey on to representative frontline workers of their organization	3
Web/Email	The e-survey was disseminated to recipients of the 3DPPE GTHA initiative via an email invitation to participate and a link to the survey. The survey form, when submitted, populated results into a database	3
Context	No websites were used in this study to advertise or list the survey	N/A
Mandatory/Voluntary	The survey was voluntary for recipients of the 3DPPE GTHA face shield donations	4
Incentives	No incentives whether monetary, prizes, or nonmonetary were offered to respondents. However, respondents were past recipients of donated 3D-printed face shields from the 3DPPE GTHA initiative	N/A
Time/Date	Our survey was open from December 1, 2020 at 12:00 pm EST to March 31, 2021 at 11:59 pm EST	4
Randomization of items or questionnaires	Items were not randomized in the survey and questions had followed a specific order	N/A
Adaptive questioning	Adaptive questioning was not utilized in this survey	N/A
Number of items	The survey consisted of 11 items	5
Number of screens (pages)	The survey questions were all found on one continuous page	N/A
Completeness check	All survey items were deemed to be mandatory, and respondents were prompted to complete outstanding items before leaving the survey page on which the item was contained	N/A
Review step	Respondents were able to change their answers prior to submission. However, they were unable to do so after submission was complete and their answers were recorded	N/A
Unique site visitor	Not applicable. Our survey was sent out by email, therefore, unique site visitors were not recorded. Unique email addresses and respondent IDs were required to identify potential duplicate entries	N/A
View rate (ratio of unique survey visitors/unique site visitors)	Not applicable. Our survey was sent out by email, therefore, unique site visitors were not recorded	N/A
Participation rate (ratio of unique visitors who agreed to participate/unique first survey page visitors)	The participation rate was 100%; 100% of respondents who agreed to participate (i.e., filled out the informed consent page) ultimately submitted the survey	N/A
Completion rate (ratio of users who finished the survey/users who agreed to participate)	A total of 63 out of 65 respondents who initiated the survey ultimately completed the survey up until the last question	6
Cookies used	Cookies were not utilized due to technical limitations of our survey platform	N/A
IP check	IP check was not utilized due to technical limitations of our survey platform to identify potential duplicate entries. Unique email addresses and respondent IDs were required to identify potential duplicate entries	N/A
Logfile analysis	Log files were not utilized due to technical limitations of our survey platform	N/A
Registration	Respondents were required to provide a valid email address and unique respondent code, provided to them by authors, to complete the survey	N/A
Handling of incomplete questionnaires	Only completed questionnaires were analyzed. Two survey responses (2/64) were incomplete such that fewer than 10% of questions were answered. The responses were removed from the survey	N/A
Questionnaires submitted with an atypical timestamp	Questionnaires where atypical timestamps were not identifiable or excludable due to technical limitations of our survey platform which did not provide time to complete data	N/A
Statistical correction	The weighting of items or propensity scores were not used to adjust scores	N/A

Appendix B: Survey questions

This survey should take five minutes to complete. Your responses are anonymous. Your name will be kept separate from your survey responses, only for the purposes of documenting informed consent.

**Table 8:** Validations and utilization of 3DP personal protective equipment: the 3DP Face Shield Research Study. 3DP: 3D printed

#	Question	Response options
1	Organization ID* You will have received this in the email along with the link to this survey	Numeric value 1–200
2	Organization type*	Multiple Choice: Hospital Department/In-Patient Healthcare Facility Urgent Care Centre or Emergency Department Outpatient Medical Clinic Long-term Care, Nursing Home or Homeless Shelter Pharmacy Allied Health Centre (PT, OT, Optom, Nutrition) Food Bank Educational or Daycare Facility First Responders Other: (free text, 100 characters)
3	Frontline worker type (Please include your discipline/subspecialty)*, e.g., physician, nurse, pharmacist, social worker, physiotherapist, optometrist, personal support worker, food bank volunteer, administrative staff, etc.	Free text, 100 characters
4	How many patients/clients do you interact with per day, on average?*	Multiple choice: 1–10, 10–50, 50–200, 200+, other (free text, 100 characters)
5	How many frontline workers does your organization have?	Multiple choice: 1–10, 10–50, 50–200, 200+, other: (free text, 100 characters)
6–15	Regarding the 3DP face shield, please rate your perceived level of*: Comfort, Stability, Visibility, Durability, Protectiveness, Ease of Assembly/Disassembly, Portability, Ability to Communicate, Compatibility With Other PPE (masks, caps, etc), Sterile Field Compatibility	Likert scale, 1 (worst) to 5 (best) or n/a
16	Relative face shield preference	Likert scale, 1 (conventional preferred), 3 (equally preferred), 5 (3DP face shield preferred)

Appendix C: Category descriptors

Note: Photographs of the 3DP and conventional face shield are provided below for your reference.

Comfort

The highest rating (5) would mean that while using the 3D printed (3DP) face shield, it did not cause you any pain over a period of your usual daily use. You may have forgotten about its presence. It did not create pressure points, pinches, sharp pricks, etc. that caused you discomfort. The lowest rating (1) would indicate the opposite, such that you would want to remove it as soon as possible due to discomfort.

Stability

The highest rating (5) would mean that the 3DP face shield barely or did not at all feel that it would fall off of your head during your usual daily use. The lowest rating (1) would indicate that you have severe, constant difficulty keeping it in place.

Visibility

The highest rating (5) would mean that the 3DP face shield barely or did not at all disrupt your ability to see your client/work environment. The lowest rating (1) would indicate that the visor blurred, distorted, or otherwise affected your ability to see clearly. Please avoid the effect of fogging as participants may have had variability in masks.

Durability

The highest rating (5) would mean that the 3DP face shield in your view was sturdy and would not “fall apart” with use over a period of days to weeks. The lowest rating (1) was that you were concerned that the 3DP face shield was fragile such that it may not stay intact or become unusable after few or multiple uses.

Protectiveness

The highest rating (5) would mean that you had the highest degree of confidence that the 3DP shield would protect you from incoming large droplets of bodily fluids. The lowest rating (1) would indicate that you had very little faith that the 3DP face shield being able to protect you from large droplets and the use of the shield would expose you to incoming large droplets of bodily fluids.

Ease of assembly/Disassembly

The highest rating (5) would mean that the assembly or the disassembly of the plastic visor component to the 3DP face shield frame was efficient and came with a low cost of time. The lowest rating (1) would indicate that it took significant effort and time to put the two components together to a level beyond practical use.

Portability

The highest rating (5) would mean that the 3DP face shield in its assembled form was easily transported for use from the supply. The lowest rating (1) indicates that the 3DP face shield was highly difficult to transport the face shield in its assembled form.

Ability to communicate

The highest rating (5) would mean that verbal communication while wearing the face shield allows you to have clear and audible conversations. The lowest rating (1) would indicate that your attempts of communication while wearing the 3DP face shield were ineffective or nonaudible.

Compatibility with other PPE (masks, caps, etc.)

The highest rating (5) would mean that you could easily put on the 3DP face shield while wearing other forms of PPE on your head such as face masks, N95 masks, or surgical scrub caps. The lowest rating (1) indicates that you struggled greatly when attempting to wear the 3DP face shield while also donning other forms of PPE.

Sterile field compatibility

The highest rating (5) would mean that you were comfortable using the 3DP face shield during a sterile procedure. The lowest rating (1) would indicate that you were highly concerned that the 3DP face shield would contaminate sterile equipment or surfaces.

Relative face shield preference

A rating of 5 would mean that, if given the choice, you would strongly prefer a 3DP face shield for your daily activities over the conventional face shield (e.g., a face shield with an elastic band and foam headband pictured below). A rating of 1 would indicate that if given the choice, you would strongly prefer a conventional face shield for your daily use over the 3DP face shield. A rating of 3 would indicate that you have no preference or that you would equally prefer either one.

## References

[1] Vordos N, Gkika DA, Maliaris G, Tilkeridis KE, Antoniou A, Bandekas DV, Ch Mitropoulos A (2020). How 3D printing and social media tackles the PPE shortage during Covid - 19 pandemic. Saf Sci.

[2] Galanis P, Vraka I, Fragkou D, Bilali A, Kaitelidou D (2021). Impact of personal protective equipment use on health care workers' physical health during the COVID-19 pandemic: a systematic review and meta-analysis [In Press]. Am J Infect Control.

[3] Bauchner H, Fontanarosa PB, Livingston EH (2020). Conserving supply of personal protective equipment-a call for ideas. JAMA.

[4] Miller DG, Pierson L, Doernberg S (2020). The role of medical students during the COVID-19 pandemic. Ann Intern Med.

[5] Wierzbicki J, Nowacki M, Chrzanowska M (2020). Additive manufacturing technologies enabling rapid and interventional production of protective face shields and masks during the COVID-19 pandemic. Adv Clin Exp Med.

[6] Pavlosky A, Glauche J, Chambers S, Al-Alawi M, Yanev K, Loubani T (2018). Validation of an effective, low cost, free/open access 3D-printed stethoscope. PLoS One.

[7] Tarfaoui M, Nachtane M, Goda I, Qureshi Y, Benyahia H (2020). 3D printing to support the shortage in personal protective equipment caused by COVID-19 pandemic. Materials (Basel).

[8] (2021). Prusa: Prusa3D - open source 3D printers by Josef Prusa. https://www.prusa3d.com/.

[9] 3DVERKSTAN: 3D-PRINTED PROTECTIVE VISOR (2021). 3DVerkstan: 3D-printed protective visor. https://3dverkstan.se/protective-visor/.

[10] Zhang PC, Ahmed Y, Hussein IM, Afenu E, Feasson M, Daud A (2020). Optimization of community-led 3D printing for the production of protective face shields. 3D Print Med.

[11] Burns KE, Kho ME (2015). How to assess a survey report: a guide for readers and peer reviewers. CMAJ.

[12] Eysenbach G (2004). Improving the quality of Web surveys: the Checklist for Reporting Results of Internet E-Surveys (CHERRIES). J Med Internet Res.

[13] Sapoval M, Gaultier AL, Del Giudice C (2020). 3D-printed face protective shield in interventional radiology: evaluation of an immediate solution in the era of COVID-19 pandemic. Diagn Interv Imaging.

[14] Spoorthy MS, Pratapa SK, Mahant S (2020). Mental health problems faced by healthcare workers due to the COVID-19 pandemic-a review. Asian J Psychiatr.

[15] Aranaz-Andrés JM, McGee-Laso A, Galán JC, Cantón R, Mira J, On Behalf Of The Team Of Work Covid- (2021). Activities and perceived risk of transmission and spread of SARS-CoV-2 among specialists and residents in a third level university hospital in Spain. Int J Environ Res Public Health.

[16] Zastrow M (2020). Open science takes on the coronavirus pandemic. Nature.

